# VirS, an OmpR/PhoB subfamily response regulator, is required for activation of *vapA* gene expression in *Rhodococcus equi*

**DOI:** 10.1186/s12866-014-0243-1

**Published:** 2014-10-03

**Authors:** Tsutomu Kakuda, Takuya Hirota, Tatsuya Takeuchi, Hirofumi Hagiuda, Shiko Miyazaki, Shinji Takai

**Affiliations:** Laboratory of Animal Hygiene, School of Veterinary Medicine, Kitasato University, Higashi 23-35-1, Towada, Aomori 034-8628 Japan

**Keywords:** Opportunistic infections, *Rhodococcus equi*, *Rhodococcus equi* VapA protein virulence, VirS

## Abstract

**Background:**

*Rhodococcus equi* is an important pulmonary pathogen in foals and in immunocompromised individuals. Virulent *R. equi* strains carry an 80-90 kb virulence plasmid that expresses the virulence-associated protein A (VapA). VapA expression is regulated by temperature and pH. The LysR-type transcriptional regulator, VirR, is involved in the regulation of the *vapA* gene. To examine the mechanism underlying transcriptional regulation of *vapA*, we characterized an *R. equi* mutant in which another putative transcriptional regulator encoded on the virulence plasmid, VirS, was deleted.

**Results:**

Deletion of *virS* reduced *vapA promoter activity* to non-inducible levels. Complementary expression of VirS in the *virS* deletion mutant restored transcription at the P_*vapA*_ promoter, even under non-inducing conditions (30°C and pH 8.0). In addition, VirS expression increased P_*vapA*_ promoter activity in the absence of functional VirR. Further, transcription of the *icgA* operon containing *virS* was regulated by pH and temperature in the same manner as *vapA*.

**Conclusions:**

This study suggests that VirS is required for VapA expression and that regulation of P_*vapA*_-promoter activity may be achieved by controlling VirS expression levels.

**Electronic supplementary material:**

The online version of this article (doi:10.1186/s12866-014-0243-1) contains supplementary material, which is available to authorized users.

## Background

*Rhodococcus equi* is a Gram-positive bacterium and a facultative intracellular pathogen of alveolar macrophages. *Rhodococcus equi* can cause bronchopneumonia in foals up to five months of age [1,2]. This bacterium has further been identified as an opportunistic pathogen in individuals compromised by immunosuppressive drug therapy, lymphoma, or acquired immunodeficiency syndrome (AIDS) [3-6].

Isolates from pneumonic foals possess a large plasmid that varies in size from 80 to 90 kb [7-9]. This plasmid is present in most clinical *R. equi* isolates recovered from infected foals but it is absent from most environmental strains [10]. Importantly, plasmid-cured isogenic mutants of virulent strains lose their ability to survive in macrophages and are unable to cause pneumonia in foals [11-14]. A highly immunogenic 15–17 kDa protein of unknown function, designated as virulence-associated protein A (VapA), is encoded within a pathogenicity island of this virulence plasmid [15]. VapA is essential for intracellular growth in macrophages and for full virulence in an infected mouse model [16].

The expression of *vapA* is controlled by temperature and pH, where maximum expression occurs at 34–41°C with a pH of 5.0 [17,18]. These characteristics suggest that *vapA* expression is intracellularly upregulated in the mammalian host. Indeed, transcription of *vapA* is increased in ex vivo murine and equine macrophages [19]. Furthermore, expression of VapA can be detected in macrophages recovered from pulmonary lesions of infected foals [20].

The *virR* gene encodes a LysR-type transcriptional regulator that affects *vapA* gene expression [21]. DNA binding studies have shown that VirR binds to a DNA fragment that contains the *vapA* promoter (P_*vapA*_). VirR alone can induce *vapA* expression, but VapA expression is enhanced when four genes downstream of *virR* are also present. One of these genes is *virS*; it encodes a protein that shares homology with the OmpR/PhoB family of response regulators [22]. It has not yet been demonstrated whether VirS is involved in the regulation of the P_*vapA*_-promoter activity.

In the present study, we constructed a *virS* deletion mutant and analyzed P_*vapA*_ promoter activity using a *R. equi* strain that harbored a P_*vapA*_*-lacZ* fusion virulence plasmid. Our results suggest that VirS contributes to the regulation of *vapA* transcription, and is thus a critical component of *R. equi* virulence.

## Methods

### Bacterial strains and culture conditions

The *R. equi* ATCC33701 strain, originally isolated from a pneumonic foal, was used as the genetic background for all experiments reported in this study. *Rhodococcus equi* was routinely grown on Luria–Bertani (LB) agar at 30°C. Apramycin (60 μg/mL) was added to LB agar to select for *R. equi* growth when necessary. All *R. equi* strains were stored at −80°C in 85% LB broth/15% glycerol (vol/vol). *Escherichia coli* DH5α was grown on LB agar or in LB broth. Antibiotics were used when necessary at the following concentrations: apramycin (60 μg/mL) or ampicillin (50 μg/mL). All *E. coli* strains were stored at −80°C in 85% LB broth/15% glycerol (vol/vol). Table 1 describes all strains and plasmids used in this study.

**Table 1:** **Bacteria and plasmids used in this study**

**Bacterial species and plasmids**	**Bacterial strains and plasmid names**	**Relevant characteristics**	**Source or reference**
*R. equi*	ATCC33701	virulent strain	
	TKR255	P*vapA*-*lacZ* fusion strain of ATCC33701	This study
	TKR303	*ΔvirS* of TKR255	This study
	TKR474	*virRΔHTH* of TKR255	This study
*E. coli*	DH5α	*F-, Φ80dlacZΔM15, Δ(lacZYA-rgF)U169, deoR, recA1, endA1, hsdR17(rK-, mK+), phoA, supE44, λ-, thi-1, gyrA96, relA1*	
plasmid	pBluescript	Amp^r^	
	pTKR131	pBluescript::*aac(3)IV*	This study
	pECO101	*E. coli-C. jejun*i shuttle vector	[23]
	pTKR144	pTKR131::*oriT*	This study
	pTKR159	pTKR144::P*aphII*	This study
	pDelta	pTKR159::*codA-upp*	This study
	pINT	pUC57::aac(3)IV-integrase	This study
	pGEM-T Easy	Amp^r^	Promega
	pTKR130	pGEM, 3.5 kb fragment containg *vapA*	This study
	pTKR139	*ΔvapA* (codon4-189) of pTKR130	This study
	pTKR148	pTKR139::*lacZ*	This study
	pTKR169	pDela::P*vapA*-*lacZ*	This study
	pTKR333	pGEM, 3.9 kb fragment containg *virR*	This study
	pTKR223	pGEM, 3.9 kb fragment containg *virS*	This study
	pTKR361	*vapRΔHTH*(codon2-50) of pTKR333	This study
	pTKR226	*ΔvirS* (codon2-252) of pTKR223	This study
	pTKR265	pDelta::*vapRΔHTH*	This study
	pTKR391	pDelta::*ΔvirS*	This study
	pTKR174	pGEM::P*aphII*	This study
	pTKR340	pGEM::P*aphII*-*virS*	This study
	pTKR344	pINT::P*aphII*-*virS*	This study
.	pTKR445	pINT::P*aphII*-*virS*D57A	This study
	pTKR509	pGEM::P*virR-VirR*	This study
	pTKR528	pINT::P*virR-VirR*	This study

### Western blot analysis

Cell extracts were boiled for 5 min in sodium dodecyl sulfate (SDS) solution (62.5 mM Tris–HCl [pH 6.8], 10% [vol/vol] glycerol, 2% [wt/vol] SDS, 5% [vol/vol] 2-mercaptoethanol, and 0.02% [wt/vol] bromophenol blue). SDS-polyacrylamide gel electrophoresis was performed using a 15% polyacrylamide gel according to the method described previously by Laemmli [24]. After electrophoresis, proteins were transferred to a nitrocellulose membrane (Protoran; GE Healthcare, Piscataway, NJ, USA), according to the manufacturer’s instructions. A monoclonal antibody against VapA (Mab10G5) was used for immunoblotting procedures [25].

### Plasmid construction

To construct mutants with unmarked in-frame gene deletions within *R. equi*, a plasmid containing the *codA-upp* cassette was constructed to facilitate positive selection of targeted gene deletion mutants. Briefly, an apramycin resistance gene [aac(3)IV] was synthesized and cloned into pUC57at the *Eco*RI and *Hin*dIII sites. Next, the apramycin resistance gene cassette was excised by digestion with *Eco*RI and *Hin*dIII, then cloned into pBluescript II SK(+) digested with *Eco*RI and *Hin*dIII to create pTKR131. *oriT* was amplified from pEco101 by polymerase chain reaction (PCR) using primers oriT-F and oriT-R. The PCR product was digested with *Spe*I and *Eco*RI, and cloned into pTKR131 digested with *Spe*I and *Eco*RI to create pTKR144. The *aphII* promoter (P_*aphII*_) region was amplified using primers aph2-F and aph2-R. The amplified DNA fragment was digested with *Hin*dIII and *Cla*I, and cloned into pTKR144 digested with *Hin*dIII and *Cla*I to create pTKR159. Finally, the *codA-upp* cassette was excised from pORF-*codA-upp* (InvivoGen, San Diego, CA, USA) by digesting with *Nco*I and *Hin*dIII, and then cloned into pTKR159 digested with *Nco*I and *Hin*dIII to create pDelta. Primers used in this study are listed in Additional file 1: Table S1.

A plasmid containing the *Streptomyces* ϕC31 integrase gene was constructed to generate the integration vector for the complementation experiments [26]. The ϕC31 integrase gene flanked by *Apa*I sites was synthesized and cloned into pUC57 digested with *Eco*RV. The ϕC31 integrase gene was excised with *Apa*I and cloned into *Apa*I-digested pTKR131 to create pINT.

### Construction of a vapA::lacZ fusion R. equi ATCC33701 strain

To construct the transcriptional fusion product containing the *vapA* promoter and the *lacZ* open reading frame (ORF), the primer pair vapA-LF and vapA-LR was designed according to the published sequence of pRE701 [22] and used for PCR amplification of a 3.5 kb fragment that included approximately 1,500 nucleotides upstream and downstream of *vapA*. This fragment was cloned into the pGEM-T Easy vector (Promega, Tokyo, Japan) to create pTKR130. PCR-mediated mutagenesis was used to delete the *vapA* gene and to create *Bgl*II and *Mfe*I sites within the coding sequence, and to produce pTKR139 using pTKR130 as the template with the primer pair ΔvapA-1 and ΔvapA-2. The deleted region in the *vapA* gene comprised codons 4–189. The promoterless *lacZ* gene was excised from pORF-lacZ (InvivoGen) by digesting with *Bam*HI and *Eco*RI, and then ligated to pTKR139 digested with *Bcl*I and *Mfe*I to create pTKR148. The DNA fragment that contained the *PvapA-lacZ* fusion was excised from pTKR148 by digesting with *Eco*RI, and then ligated to *Eco*RI-digested pDelta to create pTKR169. pTKR169 was electroporated into *R. equi* ATCC33701 as described previously [27]. Transformants (single crossovers) were selected on LB agar containing apramycin (60 μg/mL). 5-Fluorocytosine (5-FC) positive selection was performed as described previously [28]. Briefly, *R. equi* transformants were inoculated into LB liquid medium and grown overnight at 30°C. 5-FC selection of double crossovers was performed by plating 100-μL aliquots of a dilution series [10^−1^ to 10^−3^ in mineral acetate (MM-Ac) medium] of the culture onto MM-Ac agar plates supplemented with 5-FC (100 μg/mL). Plates were incubated at 30°C for 2–3 days. Virulence plasmids were isolated from 5-FC-resistant and apramycin-sensitive mutants, and analyzed by digestion with *Eco*RI. Mutants that produced the expected digestion pattern were selected (Additional file 2: Figure S1). The mutated locus was further analyzed by PCR and sequencing. One mutant strain was selected, designated TKR255, and used for further characterization.

### Construction of R. equi ΔvirS and virR_ΔHTH_ strains harboring the P_vapA_-lacZ fusion

To construct in-frame *virR* and *virS* deletion mutants*,* 3.9 kb and 3.8 kb fragments including approximately 1,500 nucleotides upstream and downstream of *virR* and *virS*, respectively, were amplified by PCR using the primer pairs virR-LF and virR-LR, and virS-LF and virS-LR*.* These fragments were cloned into the pGEM-T Easy vector to create pTKR333 and pTKR223. PCR-mediated mutagenesis was employed to delete the *virR* and *virS* genes using primer pairs ΔvirR-1 and ΔvirR-2, and ΔvirS-1 and ΔvirS-2, respectively. pTKR333 and pTKR223 were used as templates to create pTKR361 and pTKR226, respectively. The deleted region in the *virR* gene comprised codons 2–50 (*virR*_*ΔHTH*_). The deleted region in the *virS* gene comprised codons 2–252. Fragments that contained *ΔvirS and virR*_*ΔHTH*_ were excised from pTKR361 and pTKR226 by *Eco*RI digestion and ligated to *Eco*RI-digested pDelta to create pTKR265 and pTKR391, respectively. pTKR265 and pTKR391 were separately electroporated into the P_*vapA*_-*lacZ* strain (TKR255), and the *ΔvirS and virR*_*ΔHTH*_ mutants (TKR303 and TKR474, respectively) were selected and confirmed as described above (Additional file 3: Figure S2 and Additional file 4: Figure S3).

### Complementation of R. equi mutants

The *virS* ORF was amplified using the primer pair virS-NcoF and virS-HindR. The PCR product was digested with *Nco*I and *Hin*dIII, and cloned into pTKR174 digested with *Nco*I and *Hin*dIII to create pTKR340. pTKR340 was digested with *Not*I and ligated to *Not*I-digested pINT to create pTKR344. pTKR344 was electroporated into TRK303. The transformants were recovered on LB agar containing 60 μg/mL apramycin. PCR-mediated mutagenesis was used to introduce point mutations into the coding sequence of *virS* in pTKR344, and pTKR445 (pINT::*virSD57A*) was produced using the primer pair virS D57A-1 and virS D57A-2. This plasmid was electroporated into TKR303. Transformants were recovered on LB agar containing 60 μg/mL apramycin.

The fragment that contained the *virR* ORF and promoter region was amplified using the primer pair PvirR-F and virR-R. The DNA fragment was cloned into the pGEM-T vector to create pTKR509. pTKR509 was digested with *Not*I and ligated to *Not*I-digested pINT to create pTKR528. pTKR528 was electroporated into TKR474. Transformants were recovered on LB agar containing 60 μg/mL apramycin.

### β-Galactosidase assays

Cells were grown overnight at 30°C in brain-heart infusion (BHI) broth with shaking. Cultures were diluted to 1: 10 with 60 mM Tris-buffered BHI, and the pH was adjusted to pH 6.5 or pH 8.0. Cultures were grown until they reached an optical density at 600 nm (OD_600_) of 0.5–0.7. Cells were washed twice with 0.9% NaCl and resuspended in 500 μL Z buffer (60 mM Na_2_HPO_4_, 40 mM NaH_2_PO_4_, 10 mM KCl, 1 mM MgSO_4_, 50 mM β-mercaptoethanol, pH 7.6) [29]. Next, cells were permeabilized by adding 20 μL chloroform and 35 μL 0.1% SDS. One hundred microliters of 13 mM 2-nitrophenyl beta-D-galactopyranoside (Sigma-Aldrich, St Louis, MO, USA) was added to each sample, followed by incubation at 28°C for 5 min. The reaction was stopped by adding 250 μL 1 M Na_2_CO_3_, and absorbance was read at 420 nm using a spectrophotometer (GENESYS 20; Thermo Fisher Scientific, Waltham, MA, USA). The activity of each sample was calculated in Miller units as follows: 1,000 × OD_420_/OD_600_ × reaction time × volume. Assays were performed in triplicate at least three times. Graphs were created using GraphPad PRISM software.

### Transcriptional analysis of the operon containing virS

Total bacterial RNA was isolated from 5 mL cultures grown to the mid-logarithmic phase (OD_600_ = 0.25). Next, 10 mL of RNAprotect Bacteria Reagent (Qiagen, Hilden, Germany) was added to the bacterial cultures, immediately mixed, and incubated for 5 min at room temperature. Cells were harvested by centrifugation for 10 min at 5,000 × *g* at 4°C. Following this, cells were resuspended in 1 mL of RLT buffer (RNeasy Mini Kit; Qiagen) and added to 0.5 mL of 0.1 mm diameter zirconia-silica beads (μT-01; TAITEC, Saitama, Japan). Samples were lysed three times for 1 min with a bead beater (TAITEC) at 4,600 rpm. Total RNA was isolated using an RNeasy RNA mini kit (Qiagen), according to the manufacturer’s instructions. To eliminate DNA contamination, RNA was treated with 10 U of RNase-free DNase for 30 min at 37°C. DNase was inactivated by incubating the mixture for 5 min at 75°C. Next, 200 ng RNA was mixed with random 6-mers and cDNA was synthesized using a PrimeScript RT–PCR kit (Takara, Tokyo, Japan), according to the manufacturer’s instructions. Real-time RT-PCR analysis was performed in a 20-μL volume that contained 1× PowerSYBR Green PCR Master Mix (Applied Biosystems, Foster City, CA, USA), 200 nM forward and reverse primers, and the sample cDNA. The primer pairs used to amplify *vapH*, *orf7*, and *virS* were vapH-RTF and vapH-RTR, orf7-RTF, and orf7-RTR, and virS-RTF and virS-RTR, respectively. Reactions were performed with StepOne Real-Time PCR System (Applied Biosystems) using the following conditions: 95°C for 10 min, followed by 40 cycles at 95°C for 15 s and 60°C for 1 min. Results were normalized using 16S rRNA as a control and analyzed with the ΔΔ*CT* method. Graphs were created using GraphPad PRISM software.

## Results

### Development of a reporter system to analyze vapA gene expression

**Figure 1:**
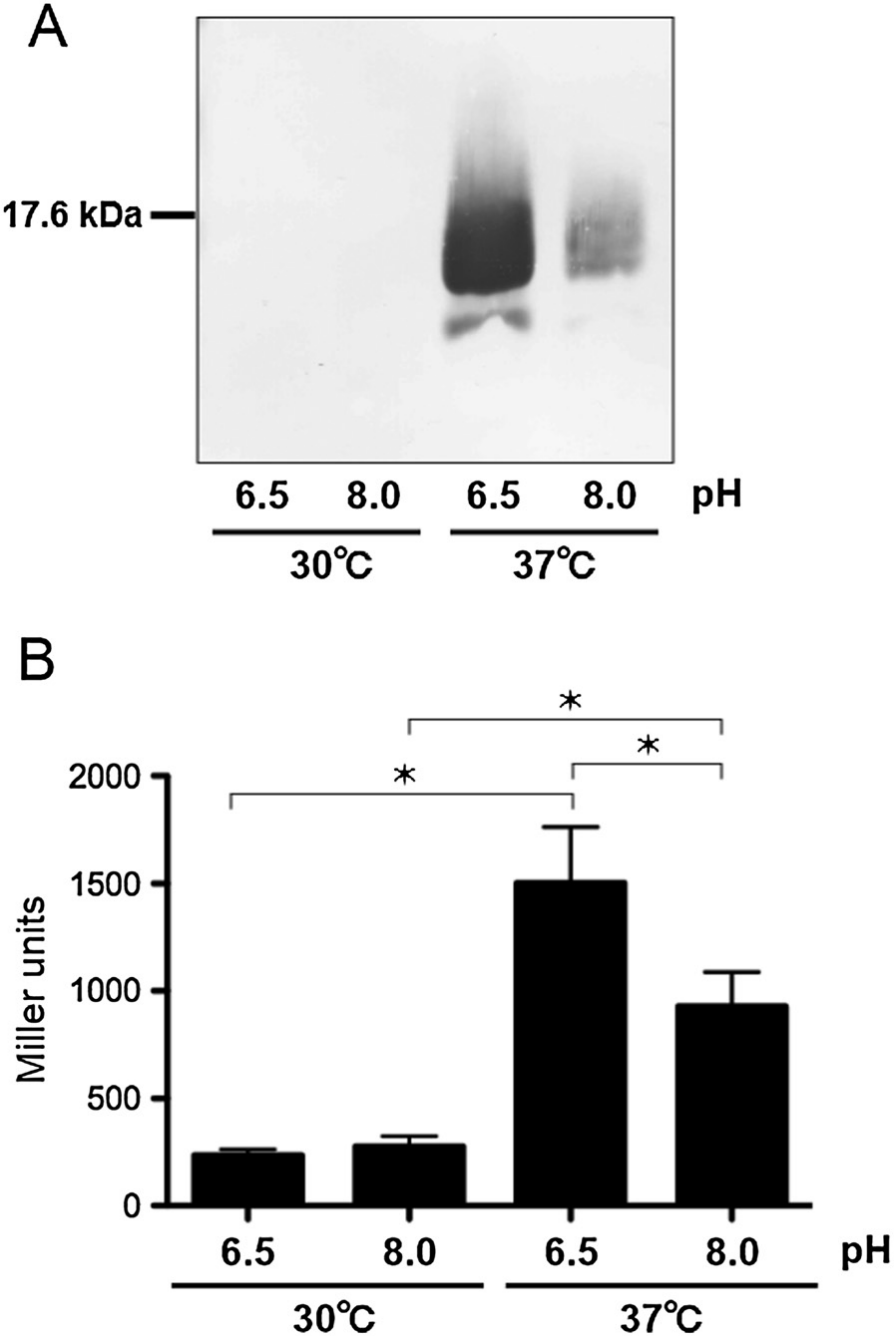
**Regulation of** ***vapA*** **transcription and VapA expression in** ***R. equi*** **. (A)** Western blot analysis performed with an anti-VapA antibody on cell extracts prepared from *R. equi* grown under conditions indicated in the Figure **(B)** β-galactosidase activity assays of P_*vapA*_ *–lacZ* fusions in the *R. equi* TKR255 strain grown at different temperatures and pH indicated in the Figure TKR255 was grown overnight at 30°C, diluted to an OD_60th0_ of 0.05 in fresh medium at the pH indicated, and incubated at the temperature indicated. β-galactosidase activity was measured in Miller units and error bars represent standard deviations for each data set (n = 3). Data were evaluated for statistical significance using one-way ANOVA followed by the Dunnett’s multiple comparison test, *p < 0.001.

Previous studies have reported that *vapA* gene expression is regulated by temperature and pH [17,18]. To confirm these regulatory effects, we performed western blot analysis on cellular extracts of the *R. equi* ATCC33701 strain grown under four different conditions: 30°C at pH 6.5, 30°C at pH 8.0, 37°C at pH 6.5, and 37°C at pH 8.0 (Figure 1A). Maximal expression of VapA occurred at 37°C and pH 6.5. At 37°C and pH 8.0, VapA expression was lower but still detectable. When bacteria were grown at 30°C; however, VapA expression was undetectable. To measure the promoter activity of the *vapA* gene using a β-galactosidase assay, we constructed a mutant strain wherein the virulence plasmid contained a P_*vapA*_*-lacZ* fusion. The highest β-galactosidase activity was detected when this strain was grown at 37°C and pH 6.5 (Figure 1B). At 37°C and pH 8.0, β-galactosidase activity was lower. Thus, these results agreed with the results of the western blot analysis. At 30°C, β-galactosidase activity was approximately 12-fold lower than that at 37°C and pH 6.5. Importantly, these results indicated that this reporter strain could be used to analyze *vapA* gene expression.

### VirS is required for vapA expression

**Figure 2:**
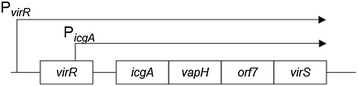
**Schematic of the** ***virR*** **and** ***icgA*** **operons.** Open boxes represent ORFs. Arrows indicate the transcript and its orientation.

**Figure 3:**
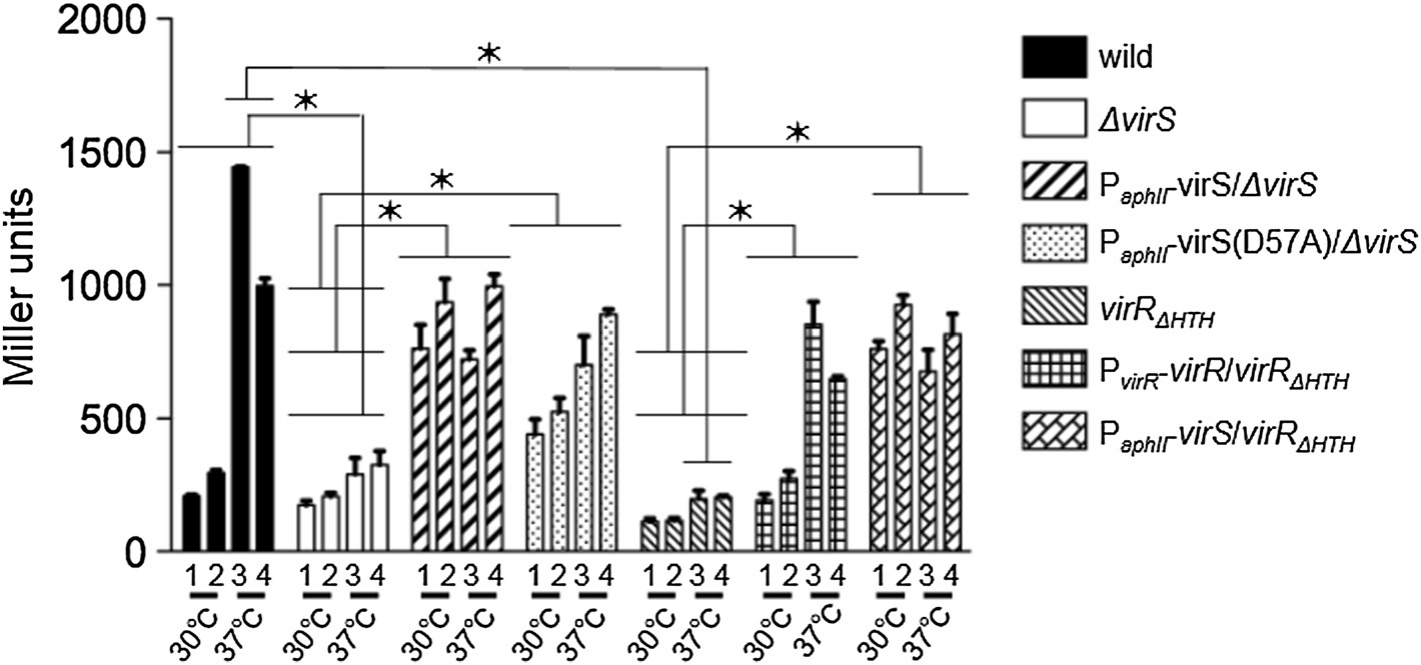
**β-Galactosidase activity assays of P**_***vapA***_ ***-acZ virS*** **and** ***virR*** **deletion mutant strains.** β-Galactosidase activity (measured in Miller units) of the wild-type strain (TKR255), *ΔvirS*, pINT::P_*aphII*_-*virS*-integrated *ΔvirS* (P_*aphII*_-virS/*ΔvirS*), pINT::P_*aphII*_-*virS (D57A)*-integrated *ΔvirS* [P_*aphII*_-*virS (D57A)*/*ΔvirS*], *virR*_*ΔHTH*_, pINT::P_*virR*_-*virR*-integrated *virR*_*ΔHTH*_ (P_*virR*_-virR/*virR*_*ΔHTH*_), and pINT::P_*aphII*_-*virS*-integrated *virR*_*ΔHTH*_ (P_*aphII*_-virS/*virR*_*ΔHTH*_) strains grown at pH 6.5 (lanes 1 and 3) or pH 8.0 (lanes 2 and 4). Error bars represent standard deviations for each data set (n = 3). Data were evaluated for statistical significance using one-way ANOVA followed by the Dunnett’s multiple comparison test, *p < 0.001.

A previous study reported that VapA expression was higher when four genes including *virS* were present in addition to *virR*, when compared with *virR* alone (Figure 2) [21]. To examine whether this increase could be attributed to VirS, the *virS* gene was deleted from the virulence plasmid in the P_*vapA*_*-lacZ* fusion strain. In the *ΔvirS* mutant, *vapA* promoter activity was reduced to a non-detectable level (Figure 3). Complementation of the *ΔvirS* mutant with *virS* expressed via from the P_*aphII*_ promoter on the bacterial chromosome increased P_*vapA*_*-lacZ* expression by more than two-fold when this strain was grown under inducing conditions. Moreover, when the complemented mutant was grown at 30°C (under non-inducing conditions), the transcription level of P_*vapA*_*-lacZ* was the same as that of the strain grown under inducing conditions. These results suggest that VirS is required for *vapA* expression and that *vapA* transcription can be induced if VirS is expressed, even when grown under non-inducing conditions.

### Phosphorylation of Asp57 is not required for the function of VirS

**Figure 4:**
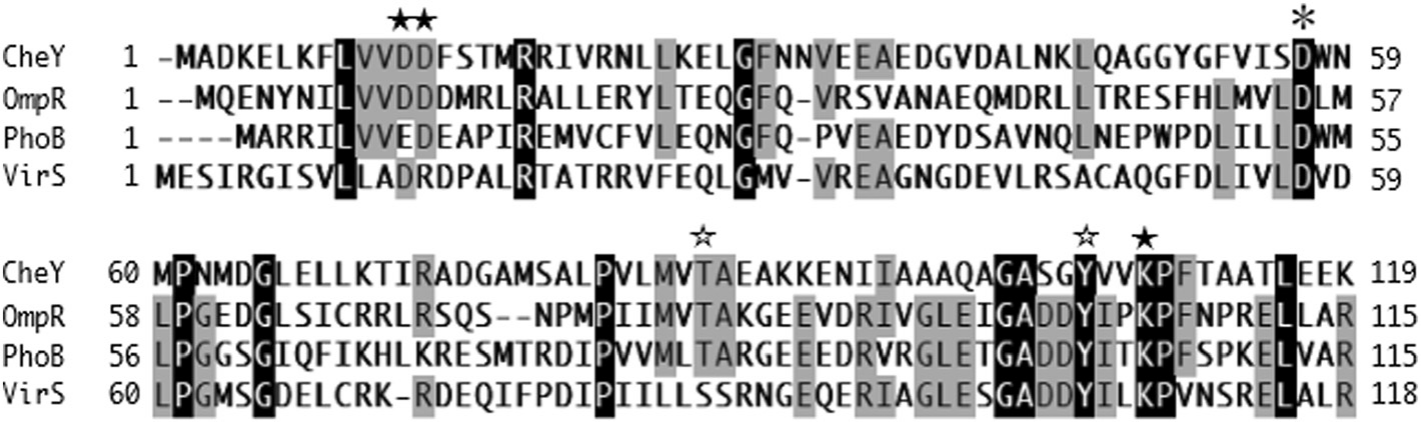
**Primary sequence alignment of VirS and other OmpR/PhoB subfamily members (** ***E. coli*** **CheY, PhoB, and OmpR).** Similarity between homologous proteins is highlighted by differences in shading: black, all amino acids in a column are identical; light gray, over half of the amino acids in a column are identical. The putative phosphorylation site (Asp57), conserved catalytic residues (Asp12, Asp13, and Lys109), and conserved conformational switch residues (Thr87 and Tyr106) are indicated by an asterisk, closed stars, and open stars, respectively. Proteins were aligned according to GENETYX-MAC software.

Activation of OmpR/PhoB family response regulators requires phosphorylation of a conserved aspartate residue [30]. Interestingly, VirS contains an aspartate residue (Asp57) that represents a putative phosphorylation site (Figure 4). To determine whether Asp57 in VirS is necessary for VirS function, Asp57 was replaced with alanine via site-directed mutagenesis. Transcription of P_*vapA*_*-lacZ* in the *virS* (Asp57Ala) mutant was comparable to that of the strain expressing wild-type VirS when they were both grown under inducing conditions (Figure 3). These results suggest that the putative phosphorylation site Asp57 is not necessary for VirS function.

### VirS can increase P_vapA_ promoter activity in the absence of a functional VirR

Previous reports suggest that VirR is required for the expression of both *vapA* and *virS* [21,31], and our results support the hypothesis that VirS can increase *vapA* expression in the presence of *virR*. To examine whether VirS function is VirR dependent, we constructed a *virR* deletion mutant*.* As the promoter of the *icgA* operon containing *virS* is located within the *virR* ORF (Figure 2), we did not delete the entire *virR* gene. Instead, only the locus that corresponded to the helix-turn-helix region (codons 2–50) was deleted and the promoter of the *icgA* operon was kept intact. The *virR*_*ΔHTH*_ mutation reduced the transcription level of P_*vapA*_-lacZ to undetectable levels under non-inducing conditions (Figure 3). When *virS* was expressed from the chromosomal P_*aphII*_ promoter in the *virR*_*ΔHTH*_ mutant, the P_*vapA*_ promoter was activated to comparable levels detected in the presence of *virR*. These results suggest that VirS can activate transcription of the P_vapA_ promoter in the absence of VirR.

### Transcription of the icgA operon is regulated by temperature and pH

**Figure 5:**
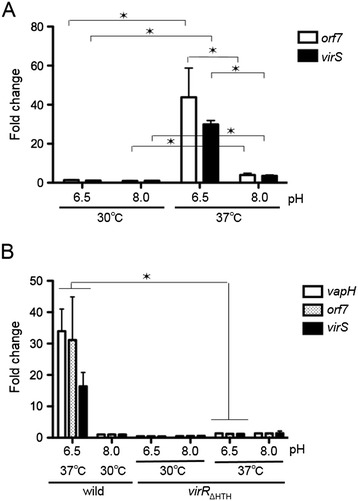
**Effects of temperature and pH on P**_***icgA***_ **promoter activity.** mRNA isolated from *R. equi* wild-type **(A)** and *virR*_*ΔHTH*_ **(B)** strains were grown under the conditions indicated, followed by semi-quantitative analysis of transcription using real-time RT-PCR. Values represent the fold increase in expression relative to expression in the wild-type grown at 30°C and pH 8.0. Data were evaluated for statistical significance using one-way ANOVA followed by the Dunnett’s multiple comparison test, *p < 0.001.

A previous study reported that transcription of the icgA operon (Figure 2) was VirR dependent and was induced at 37°C and pH 6.5 [31]. Although inducing (37°C at pH 8.0) and non-inducing (30°C at pH 6.5) conditions were compared, it was still not clear whether the transcription of the *icgA* operon was regulated by temperature, pH, or both. Thus, we semi-quantitatively determined the transcriptional level of the *icgA* operon by real-time RT-PCR. As shown previously, transcription of all the genes in this operon was induced when the wild-type strain was grown at 37°C and pH 6.5 (Figure 5A). However, their transcriptional levels were lower when cells were grown at 37°C and pH 8.0. Transcription was even lower when cells were grown at 30°C. In agreement with the previous study, we did not observe an increase in the transcription of the *icgA* operon in the *virR*_*ΔHTH*_ mutant (Figure 5B). These results demonstrate that transcription of the *icgA* operon is VirR dependent and that it is regulated by both temperature and pH.

## Discussion

Pneumonia-causing virulence by the bacterial pulmonary pathogen *R. equi* has not been fully elucidated. However, a previous study has demonstrated that VirR is involved in the regulation of the *vapA* gene located on the *R. equi* virulence plasmid [21]. To examine the contribution of *virS* (located downstream of *virR*) to *R. equi* virulence and the expression of VapA, we constructed an *R. equi virS* deletion strain paired with a P_*vapA*_*-lacZ* reporter virulence plasmid. In the current study, we demonstrate that VirS is another transcriptional regulator encoded on the virulence plasmid that is required for *vapA* transcription.

With this work, we show that deletion of *virS* reduced the transcriptional activation of the *vapA* promoter to non-inducible levels. Further, previous studies have demonstrated that *virR* is constitutively expressed and that VirS has no effect on the transcription of *virR* [31]. Together, these data suggested that VirR expression would be unaltered in a *ΔvirS* mutant, and that VirR alone is not sufficient to activate the transcription of *vapA* in the absence of VirS. In addition, deletion of *virR* completely abolished *vapA* promoter activity. However, it has previously been demonstrated that VirR is required for VirS expression [31]. Thus, we hypothesized that VirS has an indirect effect on *vapA* gene transcription. Indeed, VirS expression via the P_*aphII*_ promoter restored expression of the P_*vapA*_-*lacZ* fusion plasmid in the *virR*_*ΔHTH*_ mutant, thereby supporting this hypothesis. Furthermore, when VirS was expressed via the P_*aphII*_ promoter, there was no difference in transcription from the *vapA* promoter in the presence or absence of VirR. These results indicate that VirS can activate *vapA* transcription via the *vapA* promoter in the absence of functional VirR.

Interestingly, chromosomal integration of the P_*aphII*_-*virS* fusion did not restore P_*vapA*_*-lacZ* expression to wild-type levels in the *ΔvirS* and *virR*_*ΔHTH*_ deletion mutants. Furthermore, activation of the P_*vapA*_ promoter was observed under non-inducing conditions. It is possible that expression of the P_*aphII*_-*virS* fusion, which was present as a monocopy on the chromosome, may have resulted in lower VirS expression levels when compared with VirS expression from the virulence plasmid, as each cell harbors two or more plasmid copies [32]. In addition, this may have caused disordered regulation when VirS was expressed from the P_*aphII*_ promoter but not from the original promoter found on the virulence plasmid.

VirS is an orphan response regulator, and its cognate sensor is not found on the virulence plasmid [22]. VirS can activate the P_*vapA*_ promoter under non-inducing conditions; thus, pH and temperature are unlikely to be the stimuli that are responsible for VirS activation. We further observed that the Asp57Ala mutation did not affect activity of the P_*vapA*_ promoter when compared with that of wild-type VirS under inducing conditions. These data suggest that this putative phosphorylation site is not necessary for function of this domain. Although most residues that are critical for canonical functions in response regulators are well conserved in VirS, Asp13, which chelates the Mg^2+^ necessary for aspartic acid phosphorylation, is substituted with arginine [33-35]. Substitution of this conserved residue in the *E. coli* CheY protein means that it cannot chelate Mg^2+^ effectively and phosphorylation of Asp57 is blocked [30]. However, other OmpR/PhoB subfamily members such as *Myxococcus xanthus* FrzS and *Helicobacter pylori* HP1043 retain their functional activity in the absence of phosphorylation [36-38]. Therefore, VirS may be another atypical response regulator that does not require a sensor protein for activation.

**Figure 6:**
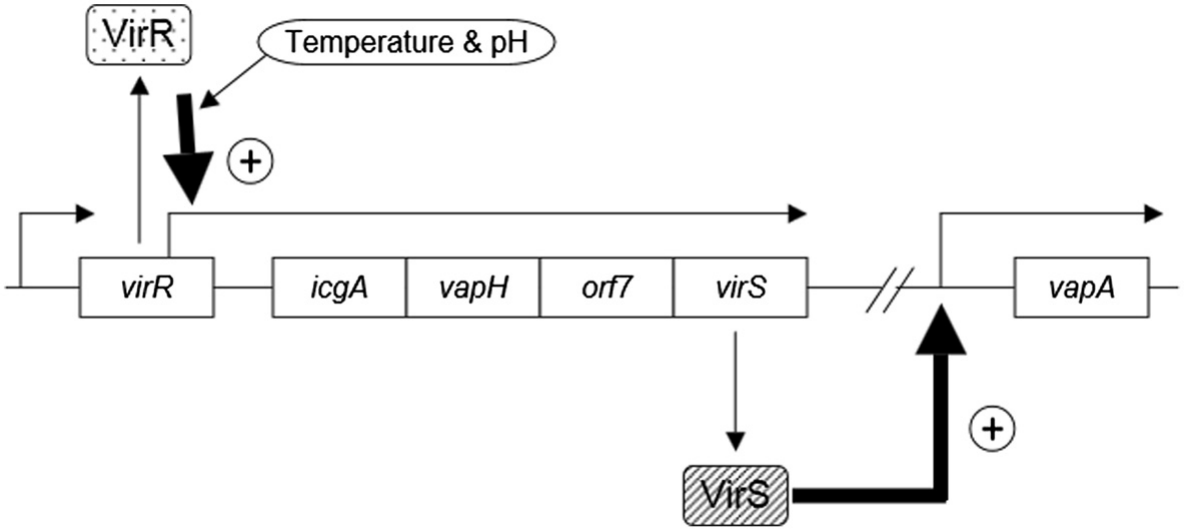
**Proposed model of** ***vapA*** **transcriptional regulation in** ***R. equi*** **.** Transcription of the *icgA* operon is VirR dependent and is regulated by temperature and pH via unknown mechanisms. VirS activates transcription of the *vapA* promoter.

In the present study, we demonstrated that the P_*icgA*_ promoter is regulated by both temperature and pH, and this corresponds to regulation of the P_*vapA*_ promoter. Further, expression of VirS from the P_*aphII*_ promoter could induce the *vapA* transcription under non-inducing conditions. These results suggest that regulation of *vapA* by temperature and pH may be achieved by controlling VirS expression levels, and a proposed model for this regulation is presented in Figure 6. As such, mechanisms that regulate expression of the *icgA* operon are likely crucial in controlling *vapA* gene expression via environmental stimuli in the bacterial pathogen *R. equi*.

## Conclusions

VirS is required for the expression of *vapA*, and VirS expression is regulated by both temperature and pH. We hypothesize that regulation of the P_*vapA*_ promoter is influenced by VirS expression levels. Future studies are required to examine the mechanisms that regulate transcription of the *virS*-containing *icgA* operon, and how this operon contributes to *R. equi* virulence.

## Additional files

Additional file 1: Table S1. Primer used in this study. Additional file 2: Figure S1. Construction of the *R. equi vapA::lacZ* fusion strain by homologous recombination. Additional file 3: Figure S2. Targeted mutagenesis of *virS* by homologous recombination. Additional file 4: Figure S3. Targeted mutagenesis of *virR* by homologous recombination.

## References

[1] Takai S (1997). Epidemiology of *Rhodococcus equi* infections: a review. Vet Microbiol.

[2] Prescott JF (1991). *Rhodococcus equi*: an animal and human pathogen. Clin Microbiol Rev.

[3] Takai S, Sasaki Y, Ikeda T, Uchida Y, Tsubaki S, Sekizaki T (1994). Virulence of *Rhodococcus equi* isolates from patients with and without AIDS. J Clin Microbiol.

[4] Takai S, Imai Y, Fukunaga N, Uchida Y, Kamisawa K, Sasaki Y, Tsubaki S, Sekizaki T (1995). Identification of virulence-associated antigens and plasmids in *Rhodococcus equi* from patients with AIDS. J Infect Dis.

[5] Takai S, Tharavichitkul P, Takarn P, Khantawa B, Tamura M, Tsukamoto A, Takayama S, Yamatoda N, Kimura A, Sasaki Y, Kakuda T, Tsubaki S, Maneekarn N, Sirisanthana T, Kirikae T (2003). Molecular epidemiology of *Rhodococcus equi* of intermediate virulence isolated from patients with and without acquired immune deficiency syndrome in Chiang Mai, Thailand. J Infect Dis.

[6] Takai S, Tharavichitkul P, Sasaki C, Onishi Y, Yamano S, Kakuda T, Tsubaki S, Trinarong C, Rojanasthien S, Sirimalaisuwan A, Tesaprateep T, Maneekarn N, Sirisanthana T, Kirikae T (2002). Identification of virulence-associated antigens and plasmids in *Rhodococcus equi* from patients with acquired immune deficiency syndrome and prevalence of virulent *R. equi* in soil collected from domestic animal farms in Chiang Mai, Thailand. Am J Trop Med Hyg.

[7] Takai S, Sekizaki T, Ozawa T, Sugawara T, Watanabe Y, Tsubaki S (1991). Association between a large plasmid and 15- to 17-kilodalton antigens in virulent *Rhodococcus equi*. Infect Immun.

[8] Takai S, Shoda M, Sasaki Y, Tsubaki S, Fortier G, Pronost S, Rahal K, Becu T, Begg A, Browning G, Nicholson VM, Prescott JF (1999). Restriction fragment length polymorphisms of virulence plasmids in *Rhodococcus equi*. J Clin Microbiol.

[9] Takai S, Murata N, Kudo R, Narematsu N, Kakuda T, Sasaki Y, Tsubaki S (2001). Two new variants of the *Rhodococcus equi* virulence plasmid, 90 kb type III and type IV, recovered from a foal in Japan. Vet Microbiol.

[10] Takai S, Watanabe Y, Ikeda T, Ozawa T, Matsukura S, Tamada Y, Tsubaki S, Sekizaki T (1993). Virulence-associated plasmids in *Rhodococcus equi*. J Clin Microbiol.

[11] Hondalus MK, Mosser DM (1994). Survival and replication of *Rhodococcus equi* in macrophages. Infect Immun.

[12] Wada R, Kamada M, Anzai T, Nakanishi A, Kanemaru T, Takai S, Tsubaki S (1997). Pathogenicity and virulence of *Rhodococcus equi* in foals following intratracheal challenge. Vet Microbiol.

[13] Giguere S, Hondalus MK, Yager JA, Darrah P, Mosser DM, Prescott JF (1999). Role of the 85-kilobase plasmid and plasmid-encoded virulence-associated protein A in intracellular survival and virulence of *Rhodococcus equi*. Infect Immun.

[14] Toyooka K, Takai S, Kirikae T (2005). *Rhodococcus equi* can survive a phagolysosomal environment in macrophages by suppressing acidification of the phagolysosome. J Med Microbiol.

[15] Sekizaki T, Takai S, Egawa Y, Ikeda T, Ito H, Tsubaki S (1995). Sequence of the *Rhodococcus equi* gene encoding the virulence-associated 15–17-kDa antigens. Gene.

[16] Jain S, Bloom BR, Hondalus MK (2003). Deletion of *vapA* encoding Virulence Associated Protein A attenuates the intracellular actinomycete *Rhodococcus equi*. Mol Microbiol.

[17] Takai S, Iie M, Watanabe Y, Tsubaki S, Sekizaki T (1992). Virulence-associated 15- to 17-kilodalton antigens in *Rhodococcus equi*: temperature-dependent expression and location of the antigens. Infect Immun.

[18] Takai S, Fukunaga N, Kamisawa K, Imai Y, Sasaki Y, Tsubaki S (1996). Expression of virulence-associated antigens of *Rhodococcus equi* is regulated by temperature and pH. Microbiol Immunol.

[19] Ren J, Prescott JF (2003). Analysis of virulence plasmid gene expression of intra-macrophage and in vitro grown *Rhodococcus equi* ATCC 33701. Vet Microbiol.

[20] Madarame H, Takai S, Morisawa N, Fujii M, Hidaka D, Tsubaki S, Hasegawa Y (1996). Immunohistochemical detection of virulence-associated antigens of *Rhodococcus equi* in pulmonary lesions of foals. Vet Pathol.

[21] Russell DA, Byrne GA, O’Connell EP, Boland CA, Meijer WG (2004). The LysR-type transcriptional regulator VirR is required for expression of the virulence gene *vapA* of *Rhodococcus equi* ATCC 33701. J Bacteriol.

[22] Takai S, Hines SA, Sekizaki T, Nicholson VM, Alperin DA, Osaki M, Takamatsu D, Nakamura M, Suzuki K, Ogino N, Kakuda T, Dan H, Prescott JF (2000). DNA sequence and comparison of virulence plasmids from *Rhodococcus equi* ATCC 33701 and 103. Infect Immun.

[23] Wiesner RS, Hendrixson DR, DiRita VJ (2003). Natural transformation of *Campylobacter jejuni* requires components of a type II secretion system. J Bacteriol.

[24] Laemmli UK (1970). Cleavage of structural proteins during the assembly of the head of bacteriophage T4. Nature.

[25] Takai S, Iie M, Kobayashi C, Morishita T, Nishio T, Ishida T, Fujimura T, Sasaki Y, Tsubaki S (1993). Monoclonal antibody specific to virulence-associated 15- to 17-kilodalton antigens of *Rhodococcus equi*. J Clin Microbiol.

[26] Hong Y, Hondalus MK (2008). Site-specific integration of Streptomyces PhiC31 integrase-based vectors in the chromosome of *Rhodococcus equi*. FEMS Microbiol Lett.

[27] Sekizaki T, Tanoue T, Osaki M, Shimoji Y, Tsubaki S, Takai S (1998). Improved electroporation of *Rhodococcus equi*. J Vet Med Sci.

[28] van der Geize R, de Jong W, Hessels GI, Grommen AW, Jacobs AA, Dijkhuizen L (2008). A novel method to generate unmarked gene deletions in the intracellular pathogen *Rhodococcus equi* using 5-fluorocytosine conditional lethality. Nucleic Acids Res.

[29] Miller JH (1972). Experiments in Molecular Genetics.

[30] Bourret RB, Hess JF, Simon MI (1990). Conserved aspartate residues and phosphorylation in signal transduction by the chemotaxis protein CheY. Proc Natl Acad Sci U S A.

[31] Byrne GA, Russell DA, Chen X, Meijer WG (2007). Transcriptional regulation of the *virR* operon of the intracellular pathogen *Rhodococcus equi*. J Bacteriol.

[32] Rodríguez-Lázaro D, Lewis DA, Ocampo-Sosa AA, Fogarty U, Makrai L, Navas J, Scortti M, Hernández M, Vázquez-Boland JA (2006). Internally controlled real-time PCR method for quantitative species-specific detection and *vapA* genotyping of *Rhodococcus equi*. Appl Environ Microbiol.

[33] Djordjevic S, Stock AM (1998). Structural analysis of bacterial chemotaxis proteins: components of a dynamic signaling system. J Struct Biol.

[34] Zundel CJ, Capener DC, McCleary WR (1998). Analysis of the conserved acidic residues in the regulatory domain of PhoB. FEBS Lett.

[35] Lewis RJ, Brannigan JA, Muchová K, Barák I, Wilkinson AJ (1999). Phosphorylated aspartate in the structure of a response regulator protein. J Mol Biol.

[36] Fraser JS, Merlie JP, Echols N, Weisfield SR, Mignot T, Wemmer DE, Zusman DR, Alber T (2007). An atypical receiver domain controls the dynamic polar localization of the *Myxococcus xanthus* social motility protein FrzS. Mol Microbiol.

[37] Hong E, Lee HM, Ko H, Kim DU, Jeon BY, Jung J, Shin J, Lee SA, Kim Y, Jeon YH, Cheong C, Cho HS, Lee W (2007). Structure of an atypical orphan response regulator protein supports a new phosphorylation-independent regulatory mechanism. J Biol Chem.

[38] Schär J, Sickmann A, Beier D (2005). Phosphorylation-independent activity of atypical response regulators of *Helicobacter pylori*. J Bacteriol.

