# Supplementary figures and images for: VirS, an OmpR/PhoB subfamily response regulator, is required for activation of vapA gene expression in Rhodococcus equi
Source: BMC Microbiol. 2014 Oct 3;14:243. doi: 10.1186/s12866-014-0243-1 (PMC4190465; doi:10.1186/s12866-014-0243-1)

A.

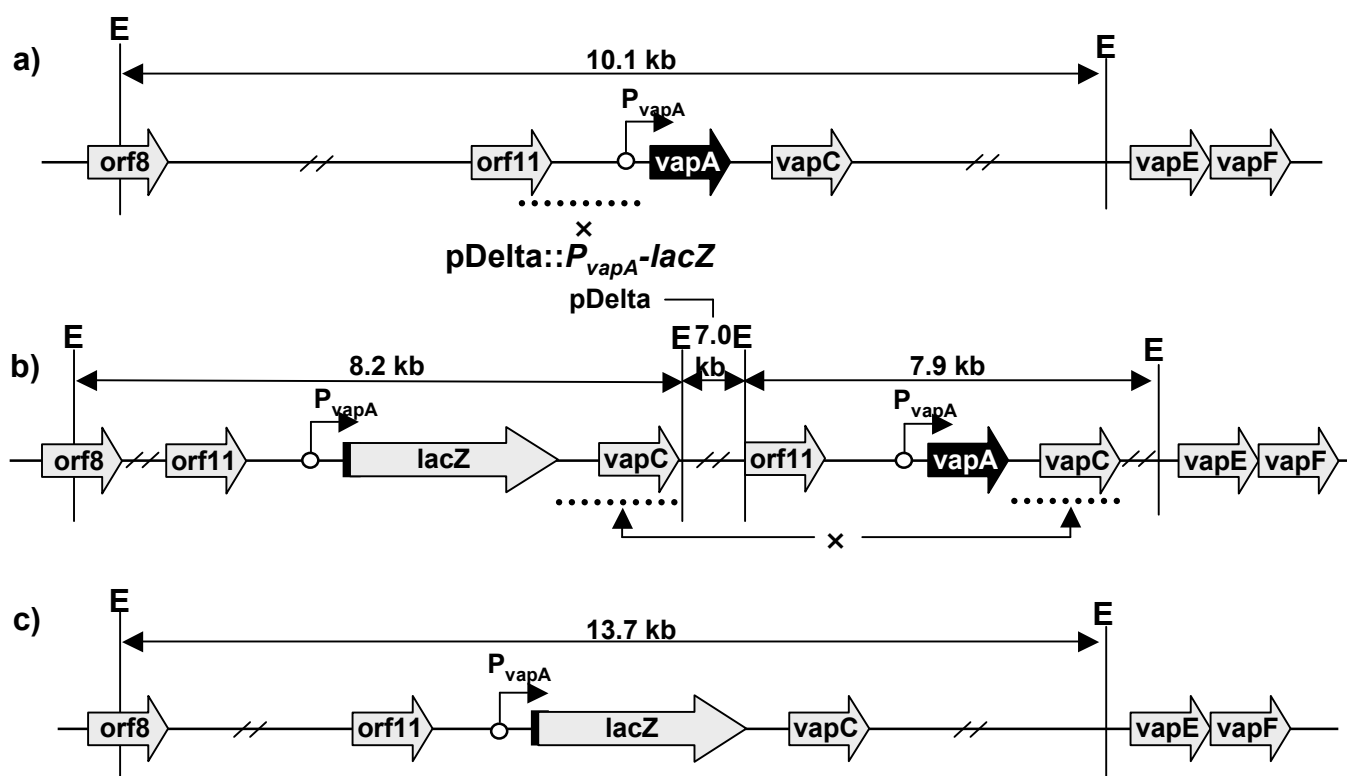

B.

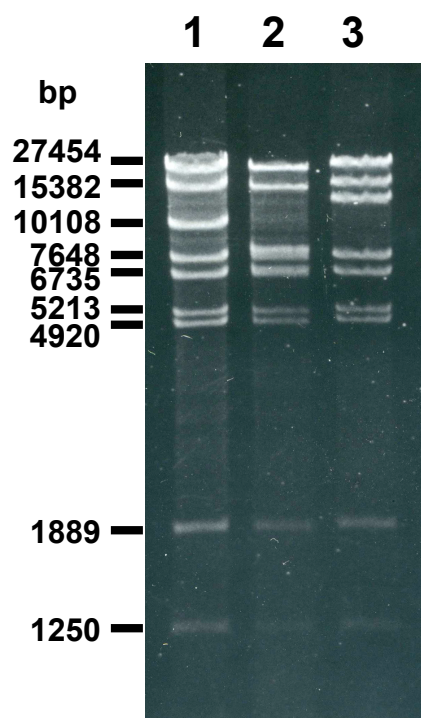

C.

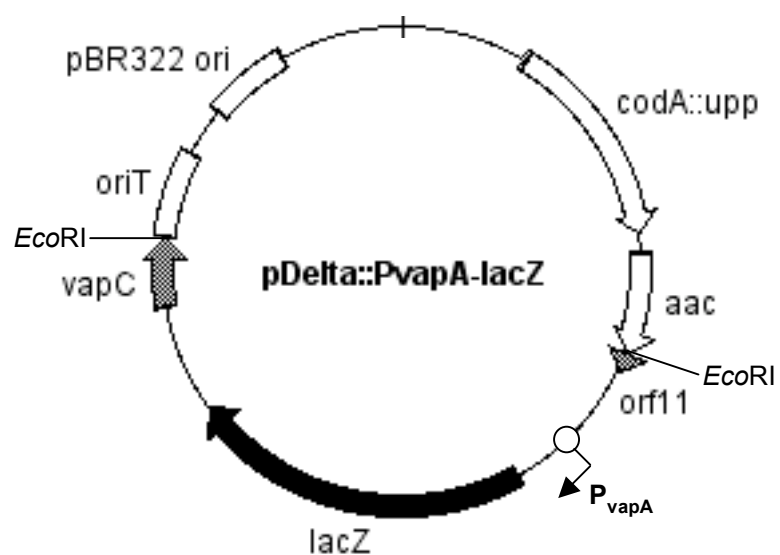

Fig.S1

Supplement: Additional file 2: Figure S1. — Construction of the R. equi vapA::lacZ fusion strain by homologous recombination. [file 12866_2014_243_MOESM2_ESM.pdf]

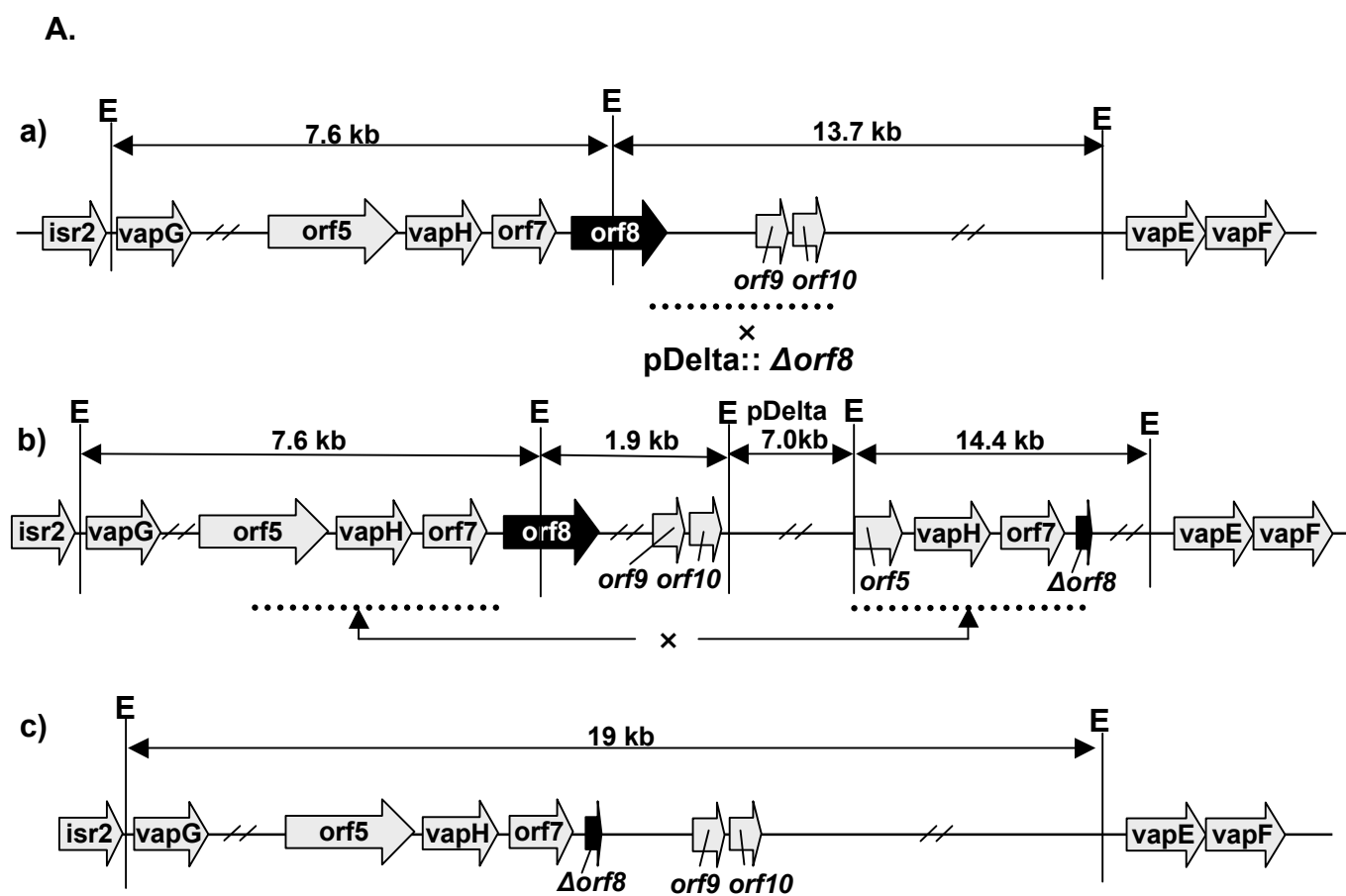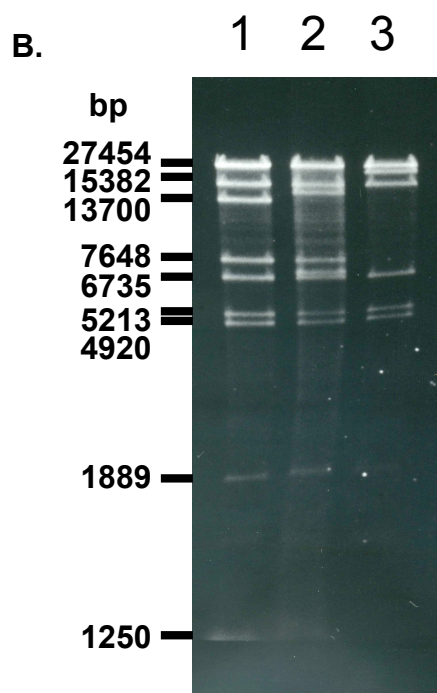

Fig.S2

Supplement: Additional file 3: Figure S2. — Targeted mutagenesis of virS by homologous recombination. [file 12866_2014_243_MOESM3_ESM.pdf]

A.

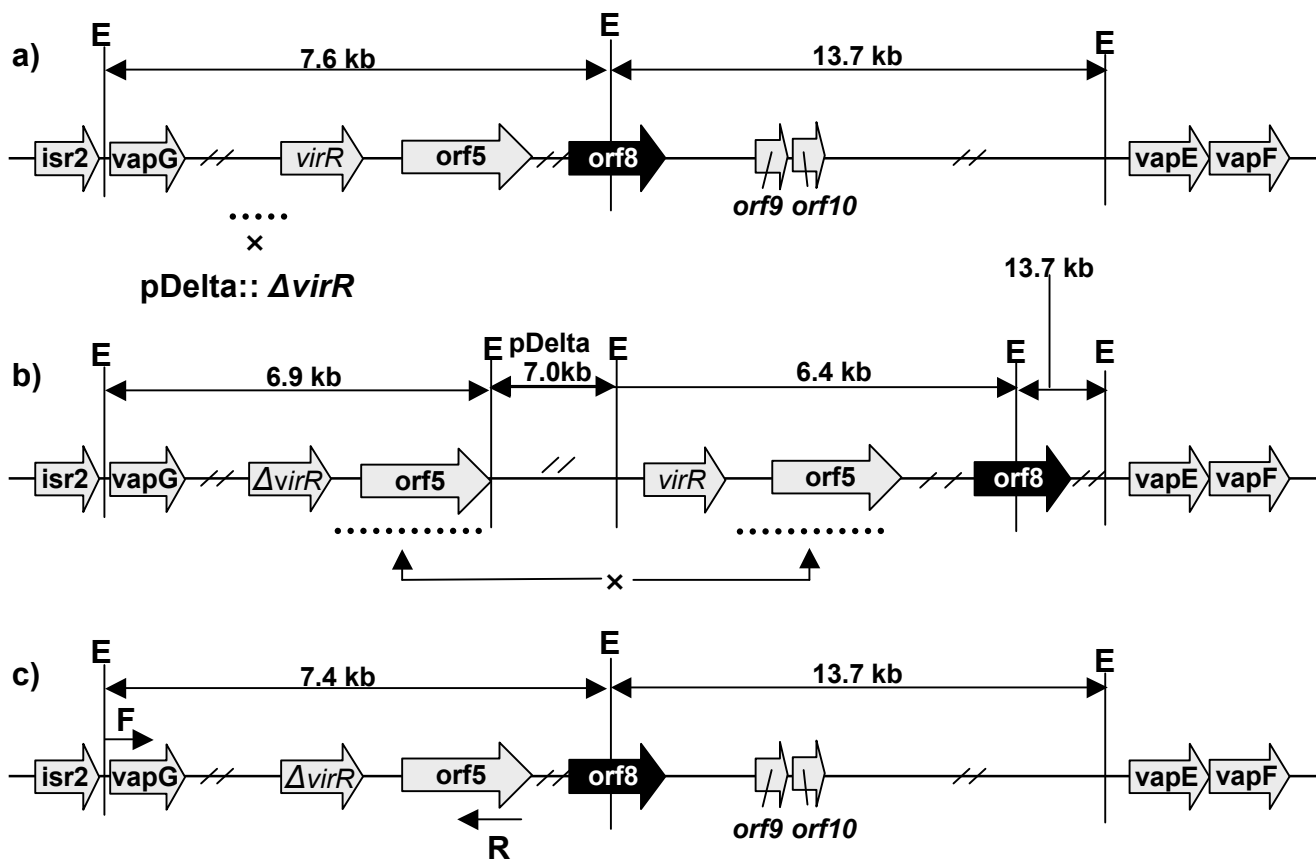

B.

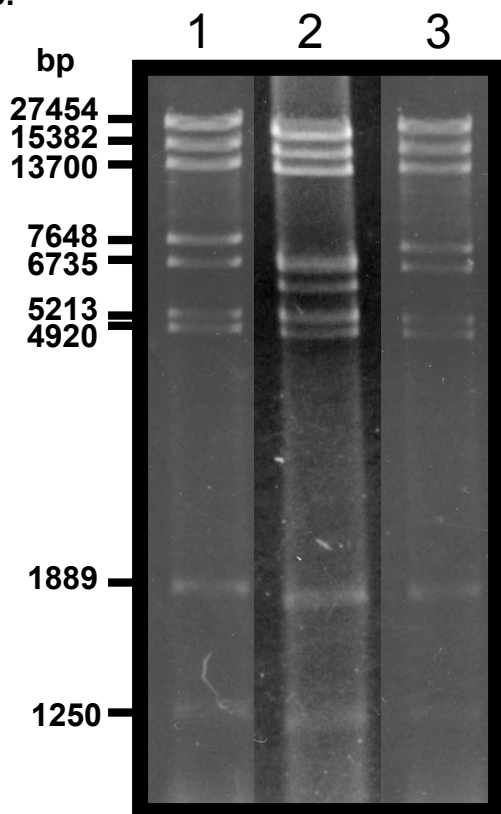

C.

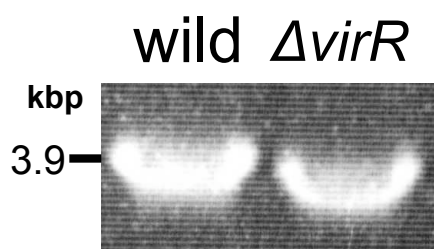

Fig.S3

Supplement: Additional file 4: Figure S3. — Targeted mutagenesis of virR by homologous recombination. [file 12866_2014_243_MOESM4_ESM.pdf]
